# Microphysiological systems as models for immunologically ‘cold’ tumors

**DOI:** 10.3389/fcell.2024.1389012

**Published:** 2024-04-22

**Authors:** Daniela Gaebler, Stephanie J. Hachey, Christopher C. W. Hughes

**Affiliations:** ^1^ Molecular Biology and Biochemistry, University of California, Irvine, Irvine, CA, United States; ^2^ Biomedical Engineering, University of California, Irvine, Irvine, CA, United States

**Keywords:** cancer immunology, tumor microenvironment, immunosuppressive, tumor on chip, microfluidic, bioengineering, tissue engineering, therapeutic development

## Abstract

The tumor microenvironment (TME) is a diverse milieu of cells including cancerous and non-cancerous cells such as fibroblasts, pericytes, endothelial cells and immune cells. The intricate cellular interactions within the TME hold a central role in shaping the dynamics of cancer progression, influencing pivotal aspects such as tumor initiation, growth, invasion, response to therapeutic interventions, and the emergence of drug resistance. In immunologically ‘cold’ tumors, the TME is marked by a scarcity of infiltrating immune cells, limited antigen presentation in the absence of potent immune-stimulating signals, and an abundance of immunosuppressive factors. While strategies targeting the TME as a therapeutic avenue in ‘cold’ tumors have emerged, there is a pressing need for novel approaches that faithfully replicate the complex cellular and non-cellular interactions in order to develop targeted therapies that can effectively stimulate immune responses and improve therapeutic outcomes in patients. Microfluidic devices offer distinct advantages over traditional *in vitro* 3D co-culture models and *in vivo* animal models, as they better recapitulate key characteristics of the TME and allow for precise, controlled insights into the dynamic interplay between various immune, stromal and cancerous cell types at any timepoint. This review aims to underscore the pivotal role of microfluidic systems in advancing our understanding of the TME and presents current microfluidic model systems that aim to dissect tumor-stromal, tumor-immune and immune-stromal cellular interactions in various ‘cold’ tumors. Understanding the intricacies of the TME in ‘cold’ tumors is crucial for devising effective targeted therapies to reinvigorate immune responses and overcome the challenges of current immunotherapy approaches.

## 1 Introduction

Cancer ranks as the second leading cause of death worldwide and has claimed the lives of approximately 10 million people in 2020 (Sung et al., 2021; Siegel et al., 2023). As our understanding of cancer as a purely genetically driven disease has evolved, cancer is now recognized as a highly dynamic evolutionary process that involves persistent interactions between the tumor cells and the associated tumor microenvironment (TME) (Anand et al., 2008; de Visser and Joyce, 2023). The TME is a highly complex ecosystem composed of extracellular matrix (ECM), tumor cells and multiple non-cancerous cell types including endothelial cells (ECs), stromal cells such as cancer-associated fibroblasts (CAFs), pericytes, mesenchymal stromal cells (MSCs) and platelets, as well as immune cells, such as macrophages, mast cells, myeloid-derived suppressor cells (MDSCs), natural killer cells, polymorphonuclear cells, dendritic cells (DCs) and T and B lymphocytes (Wu and Dai, 2017; Baghban et al., 2020; Tan and Naylor, 2022; de Visser and Joyce, 2023). Interactions among the stromal cells themselves and with the tumor lead to the release of growth factors, cytokines, chemokines, other polypeptides, enzymes and extracellular vesicles, often containing miRNAs, that influence the tumor status via controlling the activation of various metabolic signaling pathways (Wu and Dai, 2017; Baghban et al., 2020; Fontana et al., 2021; Tan and Naylor, 2022). Hence, tumor-stromal interactions, the composition of the TME and tumor immune status all contribute to determining tumor growth, metastasis, angiogenesis and therapy resistance (McMillin et al., 2013; Fontana et al., 2021; Rodrigues et al., 2021; Xiao and Yu, 2021), thereby critically influencing the clinical outcome of cancer patients.

Immunotherapy has led to a paradigm shift in the treatment of various cancer types in recent years, whereby immune checkpoint inhibitors (ICIs) are now some of the most widely used drugs in the clinics. Unfortunately, fewer than 15% of patients respond to ICI therapy (Haslam and Prasad, 2019), as the success of this therapy is dependent on various factors such as the expression of immune checkpoint markers (i.e., PD-L1, CTLA-4 and LAG3) by the tumor cells, the TME and the infiltration of functional, tumor-killing cytotoxic T cells (Bonaventura et al., 2019a; Haslam et al., 2020; Wang et al., 2023). Particularly, the presence of cytotoxic T cells in the TME is associated with improved immunotherapy outcomes and prolonged patient survival (Piersma et al., 2007; Kmiecik et al., 2013; Williams et al., 2019; Guo et al., 2023). Depending on the presence and spatial distribution of these cytotoxic T cells, one can distinguish two immunophenotypes, namely, immunologically ‘hot’ and ‘cold’ tumors (Liu et al., 2021; Wang et al., 2023). Examples of immunologically ‘cold’ tumors include ovarian, prostate, pancreatic, glioma and some renal cell cancers, while ‘hot’ tumors are typically present in the liver, lung or bladder (Liu et al., 2021; Huang et al., 2022; Wang et al., 2023). Immune-inflamed, or immunologically ‘hot’ tumors, can be characterized by genomic instability, PD-L1 overexpression, increased IFN-γ signaling, a TME enriched in tumor-infiltrating lymphocytes (TILs) and preexisting antitumor immune responses (Hegde et al., 2016; Liu et al., 2021; Wang et al., 2023). While immunologically ‘hot’ tumors tend to be more responsive to ICI treatment, immunologically ‘cold’ tumors rarely respond to ICI monotherapy due to the absence or exhaustion of TILs (Liu et al. (2021); Herbst et al. (2014). In fact, in immune excluded ‘cold’ tumors, cytotoxic T cells accumulate around the tumor but fail to efficiently infiltrate, whereas in immune-desert ‘cold’ tumors, tumor-specific CD8^+^ T cells are completely absent from the tumor and its periphery (Liu et al., 2021). Additionally, immunologically ‘cold’ tumors are characterized by the presence of immunosuppressive cell populations such as tumor-associated macrophages (TAMs), myeloid-derived suppressor cells (MDSCs) and regulatory T cells (Tregs) (Hegde et al., 2016; Liu et al., 2021; Guha et al., 2022). Overcoming the current challenges of the aberrant adaptive immune response in ‘cold’ tumors is essential for improving the success of current therapy approaches for this class of tumors. However, the identification of novel, targeted therapies necessitates a deeper understanding of the tumor-stromal, tumor-immune and immune-stromal interactions in the TME in order to elucidate their contributions to tumor responsiveness, escape and immune status.

The process of drug discovery and development entails translating fundamental biomedical research into the creation of diagnostics and therapeutics tailored to the needs of patients. Traditionally, preclinical drug development and experimental approaches to elucidate the cellular interactions in the TME have primarily exploited both *in vitro* 2D co-culture models and *in vivo* animal models (Mak et al., 2014; Fontana et al., 2021; Rodrigues et al., 2021). However, while about 30% of drugs proceed to phase I trials after successful animal testing, fewer than 8% overall ultimately pass phase I successfully, often due to unforeseen toxicities or lack of efficacy discovered during human trials (Mak et al., 2014). This imperfect transferability of either traditional cell cultures or animal models to human disease, has limited the value of these models for the development of novel, targeted therapies for cancer patients. The causes are many: the failure to recapitulate tumor or tissue-specific architecture, failure to maintain phenotypic diversity, insufficient mechanical or biochemical signaling, failure to recapitulate cell-cell and cell-ECM interactions, failure to accurately model the perfusion of oxygen, nutrients, metabolites and drugs into the tumor mass, high costs, and complexity that is hard to deconvolve (Mak et al., 2014; Katt et al., 2016; Fontana et al., 2021; Rodrigues et al., 2021). In addition, there is a growing desire to limit the use of animals in drug testing due to ethical concerns. In contrast to all of this, novel multicellular three-dimensional (3D) *in vitro* models can bridge the gap between experimental controllability and physiological relevance via reliably reproducing tissue or tumor architecture, mimicking tissue stiffness, allowing cellular crosstalk via the integration of multiple cell types and replicating various physical, morphological, structural, mechanical and biochemical cues (Jensen and Teng, 2020; Fontana et al., 2021; Hachey et al., 2021; Rodrigues et al., 2021). Notably, these physiologically relevant platforms have previously shown promise in drug discovery and testing by faithfully recapitulating pathophysiological human drug responses (Asghar et al., 2015; Benam et al., 2016; Hachey et al., 2021); hence offering the potential to advance the development of personalized therapy approaches. Notably, 3D *in vitro* models range from cell/ECM-based assays (i.e., hydrogel-based cell-laden scaffolds and 3D bioprinted cultures) and cell-based models (i.e., spheroids and organoids) to microfluidic systems.

Microphysiological systems (MPS), or “organ-on-a-chip” devices, consist of multi-channel systems in the micrometer size range, featuring continuously perfused micro-chambers seeded with cells. In a subset of MPS, the size of the micro-channels is comparable to blood capillaries, which facilitate the transport of nutrients, metabolites and gases, enable the spatiotemporal distribution of signaling molecules and/or cells and provide physiologically relevant biomechanical stimulation in real time to reliably replicate the (patho-) physiological functions of tissues and organs (Park et al., 2019b; Baptista et al., 2022; Hachey et al., 2023a; Bouquerel et al., 2023). To date, MPS models have been developed for the majority of human tissues and organs, and have also been established to study a diverse range of malignancies, including cancer (Baptista et al., 2022; Hachey et al. (2021; 2024); Jiang et al., 2024; Haque et al., 2022; Shen et al., 2023; Flont et al., 2023; Straehla et al., 2022). Importantly, the flexible structural layout of organ chips enables the exploration of interactions among different biological compartments, encompassing cellular and extracellular matrix (ECM) interactions, parenchymal-vascular connections, and tissue-tissue or organ-organ interfaces to create “multi organ-on-a-chip” and “body-on-chip” systems (Baptista et al., 2022; Picollet-D’hahan et al., 2021). Likewise, microfluidic models have the capability to thoroughly model the tumor immune microenvironment comprised of endothelial, stromal and immune cell populations (Miller et al., 2020; Surendran et al., 2021; Hachey et al., 2023a); making them useful preclinical tools to enhance our understanding of basic tumor pathology, immune cell exclusion and immunosuppression. Furthermore, they have great potential to revolutionize the drug development process via helping us understand how to reshape the TME, which is of particular interest in immunologically ‘cold’ tumors. In this context, we review existing microfluidic models that aim to dissect tumor-stromal, tumor-immune and immune-stromal interactions in the TME of various ‘cold’ tumors.

## 2 Defining the ‘cold’ tumor microenvironment

While progress in immunotherapy has revolutionized the treatment landscape for numerous cancers, certain tumor types with an immunologically ‘cold’ phenotype remain an exception (Bonaventura et al., 2019b). Despite numerous intrinsic factors influencing tumor immunogenicity, including tumor mutational burden, mismatch repair status and gene expression profile, it is essential to acknowledge the equally significant role of the TME. The pre-existing immune landscape in the TME predicts malignancy outcomes and response to immunotherapies. As noted above, based on their TME, tumors can be categorized into two general types: ‘hot’ tumors, distinguished by an immunosupportive TME and a positive response to immunotherapy, and ‘cold’ or non-immune reactive tumors exhibiting a poor response to immunotherapy and characterized by an immunosuppressive TME (Zhang et al., 2022; Benoit et al., 2023). However, it is crucial to recognize that the distinction between immunologically ‘hot’ and ‘cold’ tumors exists along a spectrum, varying not only among different cancer types but also among individual patients within the same cancer type. Further, throughout tumorigenesis and disease progression, cancer cells may acquire phenotypic traits enabling them to evade immune recognition or destruction (Shelton et al., 2021). The immunoscore is a quantitative method for characterizing tumors by evaluating immune cell type, functionality, density, and spatial distribution in the tumor microenvironment (TME), providing prognostic insights across cancer types (Bruni et al., 2020). A high immunoscore indicates a robust immune infiltrate correlated with better clinical outcomes, while a low score may suggest a less effective anti-tumor immune response due to TIL exclusion or exhaustion. Tumors vary in immunoscore, with ‘cold’ tumors lacking immune cell infiltration, ‘hot’ tumors showing high infiltration in both core and margin, and ‘altered’ (warm) tumors having modified immune cell infiltrate in either the tumor margin alone (‘altered-excluded’) or both areas (‘altered-immunosuppressed’) (Ren et al., 2022). Recognizing context-dependent and patient-specific factors is crucial for understanding and addressing immunosuppression in tumors.

**FIGURE 1:**
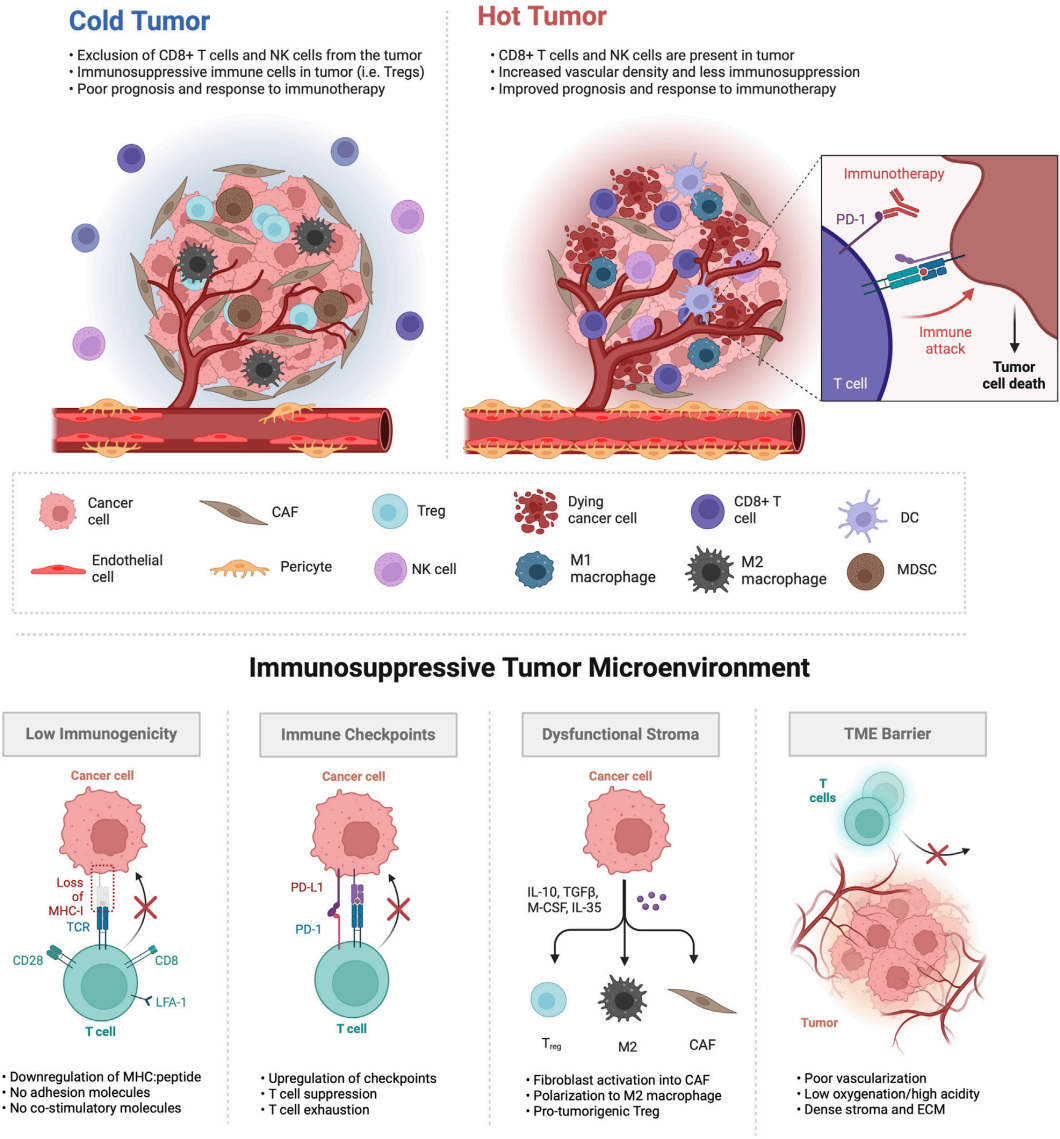
Tumor microenvironments characterized as immunologically ‘cold’ exhibit distinct features. In the upper panel, ‘cold’ tumors are often less vascularized, presenting abnormal vasculature with lack of tight endothelial junctions, inadequate pericyte coverage, increased permeability and poor perfusion. The infiltration of T regulatory cells (Tregs), myeloid-derived suppressor cells (MDSCs) and M2 polarized macrophages is prevalent in ‘cold’ tumors, as are cancer-associated fibroblasts (CAFs), while cytotoxic CD8^+^ T cells, natural killer (NK) cells, dendritic cells (DCs) and M1 macrophages are relatively excluded and/or exhausted. In contrast, ‘hot’ tumors exhibit an opposite phenotype, demonstrating responsiveness to immunotherapy and resulting in cancer cell death. The lower panel illustrates various mechanisms through which ‘cold’ tumors dysregulate immune function within the tumor TME.

As illustrated in Figure 1, ‘cold’ tumors, in contrast to ‘hot’ tumors, exhibit immune exclusion or exhaustion within a desmoplastic stroma. The TME encompasses diverse interactions with both adaptive and innate immune cells, whose functions can either suppress or support tumorigenesis. Moreover, the functions of immune cells within the TME are subject to the influence of tumor type and context. Heterogeneity in immune cell infiltrates highlights the need to investigate the mechanisms that dictate whether a tumor exhibits a ‘hot’ or ‘cold’ status. Adaptive immune cells, including T cells and B cells, respond to specific antigens and possess immunological memory. Conversely, innate immune cells employ non-specific defense mechanisms against foreign antigens, including those arising from genetic aberrations harbored by cancerous cells. This group encompasses macrophages, neutrophils, natural killer (NK) cells, and dendritic cells. Immunosuppressive cell types, such as TAMs, regulatory T cells, MDSCs, and cancer-associated fibroblasts (CAFs) play key roles in establishing an immunosuppressive environment in the TME and interfering with cytotoxic T cell activation (Wang et al., 2023; Xiao et al., 2023). TAMs, recruited to the tumor site, undergo polarization, adopting either anti-tumoral (M1) or pro-tumoral (M2) macrophage phenotypes (Ren et al., 2022). Pro-tumoral M2 macrophages contribute to angiogenesis, metastasis, and signaling pathways associated with treatment resistance. MDSCs serve as significant regulators of cancer progression by secreting cytokines like TGF-β, VEGF, and MMP9, promoting angiogenesis and metastasis. Additionally, MDSCs interact with NK cells, T cells, and macrophages, exerting immunosuppressive effects and inhibiting their activation. Moreover, MDSCs facilitate the proliferation and activation of Tregs, adding complexity to the immunosuppressive microenvironment in cancer.

‘Hot’ tumors show enrichment in CD8^+^ lymphocytes and M1 TAMs, with low infiltration of MDSCs or CAFs, resulting in a more favorable response to immunotherapy. In contrast, the TME of ‘cold’ tumors is defined by the presence of more Tregs than CD8^+^ lymphocytes and high infiltration of M2 TAMs, MDSCs, and CAFs (Benoit et al., 2023). Therefore, ‘cold’ and ‘altered’ tumors typically exhibit limited clinical benefits from immune checkpoint inhibitors. The vasculature in tumors exhibits structural disorganization and functional abnormalities, particularly in ‘cold’ tumors (Choi and Jung, 2023). ‘cold’ tumors often suffer from impaired blood perfusion, restricting the effective delivery of therapeutics and cytotoxic immune cells, leading to hypoxia—a hallmark of the abnormal TME inducing immunosuppression (Schaaf et al., 2018). Additionally, cancer cells, through the activation of oncogenic signaling pathways and the upregulation of cellular immune checkpoints such as programmed death ligand 1 (PD-L1), evade immune surveillance and exploit stromal cells to facilitate tumor progression (Van Der Woude et al., 2017). These cells, through heightened production of immunosuppressive and tumor-promoting cytokines, influence the polarization, activity, and expansion of diverse immune cell subpopulations. They also interact with ECs, inducing changes in structural integrity and functional properties, ultimately diminishing antitumor immune responses. Reduced immunogenicity, marked by decreased expression of tumor-associated antigens (TAAs) or major histocompatibility complex (MHC) class I molecules, is another mechanism contributing to an immunoevasive cancer cell phenotype (Van Der Woude et al., 2017). As a strategic approach, reprogramming these tumors into ‘hot’, inflamed states may enhance responses to immunotherapy.

Ipilimumab, the first FDA-approved monoclonal antibody targeting the negative immune checkpoint protein CTLA-4, enhances T cell activation and depletes Tregs in the tumor bed (Bagchi et al., 2021). Preclinical studies emphasizing the pivotal role of the PD-1/PD-L1 axis in suppressing effector T cell function led to FDA approval of nivolumab and pembrolizumab, monoclonal antibodies to PD-1, for various solid immunologically ‘hot’ cancers like melanoma, non-small cell lung cancer (NSCLC), and renal cell carcinoma (Bagchi et al., 2021). Clinical trials highlight their effectiveness compared to conventional chemotherapy by reactivating T cells within the tumor mass, specifically for a subgroup of patients with tumors overexpressing PD-L1 as a response to a robust and active immune infiltrate (Bruni et al., 2020). Nevertheless, there is a shortage of biomarkers capable of distinguishing between a positive response and resistance to immune checkpoint inhibitors (ICIs). Studies reveal a lack of correlation between the level of PD-L1 expression and the response to ICI treatment in certain cancers (Davis and Patel, 2019; Li et al., 2022). Despite this, research suggests that immunogenic cell death (ICD) induced by chemotherapy, targeted therapy, and/or radiotherapy not only leads to cancer cell death but also stimulates anti-tumor immune responses through the release of tumor neoantigens and damage-associated molecular patterns (DAMPs) (Zhai et al., 2023). This implies that combining multiple therapies may enhance the response rate of patients. However, the challenge persists in treating ‘cold’ tumors with ICIs (Majidpoor and Mortezaee, 2021). To address this, various immunotherapeutic strategies have emerged, including adoptive cell transfer, primarily utilizing genetically modified T cells expressing chimeric antigen receptors (CAR T-cells) to recognize and eliminate cancer cells directly (Sterner and Sterner, 2021). Adjuvant therapies, such as dendritic cell, mRNA, and peptide-based vaccinations, aim to boost the patient’s anticancer immunity by stimulating antigen-specific CD8^+^ T cells (Saxena et al., 2021). These mechanisms have the potential to convert ‘cold’ tumors into immunogenic ‘hot’ tumors by generating potent cytotoxic T lymphocyte responses capable of eradicating tumors (Choi and Jung, 2023). Additional strategies to elicit a robust immune response in these tumors involve agonistic antibodies to promote co-stimulation (e.g., via CD137 (4-1BB), OX40, and GITR), cytokine therapy (e.g., IL-2, IL-12, IFN-γ), bispecific antibodies, and oncolytic viruses (Mu et al., 2023). Furthermore, we suggest addressing TME normalization through targeted therapy, in conjunction with immunotherapy, to create a synergistic effect and overcome challenges to therapeutic delivery - ultimately amplifying the overall effectiveness of treatment. While previous references have extensively detailed each interconnected component in the immunologically ‘cold’ TME (Bonaventura et al., 2019b; Zhang et al., 2022; Benoit et al., 2023; Wang et al., 2023), our emphasis is on highlighting the critical need to accurately replicate the intricate interplay among multiple TME components in preclinical studies. The objective is to enhance the modeling of immunologically ‘cold’ tumors, aligning it more closely with the unique physiological conditions of each patient, and ultimately improve individual outcomes.

## 3 Current microphysiological systems of ‘cold’ tumors

In pursuit of human model systems with high clinical predictivity in immuno-oncology, three-dimensional microfluidic-based human tumor models, or “tumor chips”, have been developed and offer the unique advantage of observing biological phenomena with high spatiotemporal resolution. These chips incorporate cell line-derived or patient-derived cancer cells, spheroids or cancer organoids embedded in a 3D matrix, allowing the precise recapitulation of spatial aspects of *in vivo* tumor architecture (Hachey and Hughes, 2018). Tumor chips have been developed to model a broad variety of cancers, including primary lung cancer, prostate cancer, breast cancer, colorectal cancer, pancreatic cancer, melanoma, liver cancer and glioblastoma (among other cancers), as well as cancer metastasis to the bone, the brain, the liver and the lung (Hachey et al. (2021; 2024); Jiang et al. (2024); Haque et al. (2022); Shen et al. (2023); Flont et al. (2023); Straehla et al. (2022); Hsiao et al. (2009); Khazali et al. (2017); Aleman and Skardal (2019); Oliver et al. (2020)). Further, tumor chips faithfully replicate cancer cell gene expression patterns and have the potential to also model complex cytokine and chemokine signaling networks, which may influence immune cell exhaustion and therapeutic resistance (Powers et al., 2002; Holmes et al., 2011; Marushima et al., 2011; Edmondson et al., 2014; Jensen and Teng, 2020). In addition, many studies have highlighted the critical importance of the microenvironment for promoting or suppressing tumor growth, invasion, metastasis, and response to therapy (McMillin et al., 2013; Fontana et al., 2021; Rodrigues et al., 2021; Xiao and Yu, 2021). Tumor chips can capture many of these features, including cell-cell and cell-ECM interactions, mechanical forces within the ECM, central hypoxia, desmoplasia, gradients of nutrients, pH, and soluble factors (Koens et al., 2020; Miller et al., 2020; Jouybar et al., 2023). These features are often challenging to replicate in 2D cell culture, emphasizing the importance of advancements in 3D culture for unraveling the interconnected role of the TME in the progression of ‘cold’ tumors. Furthermore, 3D tumor-on-a-chip models enable the isolation and examination of the impact of individual variables, a task that is challenging in in vivo systems. Consequently, there has been a gradual increase in the complexity of these tumor chip systems, incorporating features such as flow-based perfusion and soluble factor gradients. These advancements integrating tumor vasculature, stromal components, and, more recently, immune cells, contribute to a more comprehensive understanding of the TME.

**FIGURE 2:**
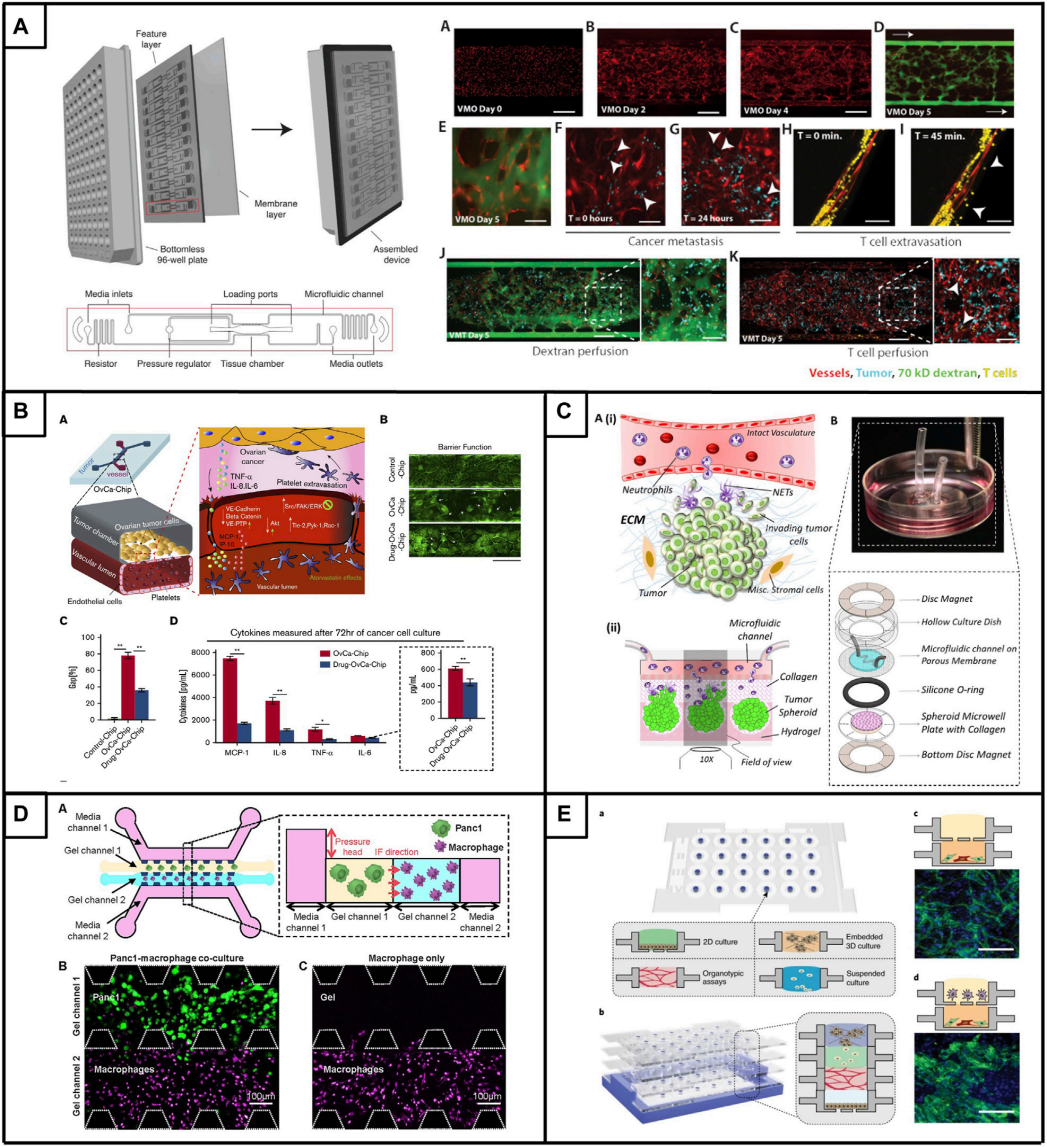
Tumor chips to model immunologically ‘cold’ tumors. **(A).** The vascularized micro-tumor (VMT) is a physiologically relevant tumor chip model. Platform assembly features a PDMS layer with 12 device units bonded to a bottomless 96-well plate, sealed with a transparent polymer membrane. Vascular networks are fully perfused and can be used for onco-immunology studies by perfusing cancer cells and/or T cells. Adapted from Hachey et al. (2023a). **(B)**. **(A)** Microfluidic co-culture model of ovarian tumor cells and endothelial cells enabling the extravasation of platelets. Treatment with atorvastatin limits platelet extravasation though improvement of vessel barrier function **(B)** and endothelial gaps **(C)**, which is accompanied by changes in the expression of major inflammatory cytokines **(D)**. Adapted from Saha et al. (2020). **(C)**. (Ai) Schematic overview of the interactional dynamics between extravasating neutrophils and pre-metastatic ovarian tumor cells (Aii) Schematic conceptulization of the TIME in the microfluidic device **(B)** Assembly of microfluidic dish platform to create TIME-on-a-chip. Adapted from Surendran et al. (2021). **(D)**. **(A)** Microfluidic co-culture model design featuring PDAC tumor cells (Panc1) and macrophages with the inclusion of interstitial flow (IF). Representative confocal image of gel channels seeded with cells at 0 h, **(B)** depicting tumor cells, and **(C)** without tumor cells. Adapted from Lee et al. (Lee et al., 2020). **(E)**. Microfluidic system that allows individually prepared cell layers **(A)** to be vertically stacked **(B)** to study complex multicellular interactions. **(C)** Co-culture of endothelial cells and fibroblasts leads to the formation of micro-vessels **(D)** The multiculture of endothelial-fibroblast (bottom layer) and macrophages (upper layer) resulted in abnormal vessel morphology. Adapted from Yu et al. (2019).

The vasculature is a key component of the TME and has become essential for tumor chip models aiming to establish a physiologically relevant barrier in order to more reliably assess drug delivery, evaluate drug effectiveness, examine vascular activation and study the interaction, function and extravasation of immune cells. We have described a tumor chip that includes a functional vasculature and allows for the incorporation of immune cells (Figure 2A) (Hachey et al., 2023a). Specifically, this model allows for the creation of “vascularized micro-tumors” (VMTs), whereby the coculture of endothelial colony-forming cell-derived ECs, normal human lung fibroblasts and cancer cells under dynamic flow conditions enables the *de novo* formation of perfusable microvascular networks that surround the micro-tumors, supplying nutrients and therapeutics just as tumor-associated vessels do *in vivo* (Sobrino et al., 2016). The model has been adapted to multiple cancer types, including immunologically ‘cold’ or ‘mixed’ tumors such as breast cancer, MSS subtype colorectal cancer and ovarian cancer, which display varying degrees of vascular disruption (Jahid et al., 2022; Hachey et al., 2024), Hachey et al. (2021)). Specifically, the model serves as a modality to test drug sensitivities of primary derived cells from colorectal cancer patients (Hachey et al., 2023b). Additionally, this model has been used to demonstrate the extravasation of T cells, underlining its potential in assessing tumor-stromal, tumor-immune and immune-stromal interactions (Hachey et al., 2023a). Importantly, immune cells must cross the endothelial layer to reach the tumor, modeling *in vivo* physiological barriers to therapeutic delivery and efficacy. Besides this model, several other tumor chips have been developed, encompassing features such as tumor vasculature (Yu et al. (2019); Wan et al. (2023); Nashimoto et al. (2020); Neufeld et al. (2021); Kim et al. (2022); Mannino et al. (2017); Shirure et al. (2018), stromal cells (Yu et al. (2019); Ibrahim et al. (2022); Karakas et al. (2017), or immune cells (Surendran et al. (2021); Aung et al. (2020); Lee et al. (2018); Kim et al. (2019), and offering advantages in terms of rapid fabrication, reproducibility, real-time imaging and analysis, capturing tumor type and heterogeneity, facilitating robust drug testing, target discovery and personalized therapy screenings, and enabling multicellular crosstalk. However, only few models have reached a high level of (cellular) complexity and functionality. Enhancing our comprehension of stromal and immune processes within immunologically ‘cold’ tumors is crucial for optimizing the efficacy of therapy and immunotherapy approaches.

While still in their nascent phase of development, advanced tumor chips have been created as proof-of-concept for immuno-oncology studies, particularly in the context of immunologically ‘hot’ tumors. Comprehensive reviews have explored these advancements (Parlato et al., 2021; Paterson et al., 2021; Shelton et al., 2021; Ngan Ngo et al., 2023). These studies have evaluated diverse treatment modalities and tumor-stromal-immune interactions in nearly every tumor type, and incorporated a range of immune cell types, from various sources such as cell lines, organoids, peripheral blood mononuclear cells, and patient-derived tumor-infiltrating lymphocytes (Biselli et al., 2017; Lee et al., 2018; Boussommier-Calleja et al., 2019; Mu et al., 2023). Notably, several of these models, accompanied by the evolution of tailored designs, are now being applied to explore traditionally immunologically ‘cold’ tumors. Below, we explore the progress related to three traditionally ‘cold’ tumors (ovarian cancer, pancreatic ductal carcinoma, and prostate cancer) and one tumor now recognized as ‘mixed’ (renal cell carcinoma (RCC)), offering detailed insights into the diverse immunological states observed in immune-suppressed tumors.

### 3.1 Ovarian cancer

Ovarian cancer (OC) is the deadliest gynecological disease worldwide and ranks as the fifth leading cause of cancer-related death in the US (Garcia and Singh, 2013). For the year of 2024, the American Cancer Society estimates about 20,000 women to be newly diagnosed with ovarian cancer, while more than 12,000 ovarian cancer patients are expected to succumb due to their disease (Siegel et al., 2024). The high mortality rate of OC can be partially explained by inadequate screening techniques, late-stage disease detection and disease heterogeneity with multiple histotypes described (Christie and Bowtell, 2017; Roberts et al., 2019). High-grade serous ovarian cancers (HGSOC) are the most prevalent type of epithelial ovarian tumors (about 75%) and characterized by TP53 driver mutations and extensive genome rearrangement (Christie and Bowtell, 2017). The 5-year survival rate for these tumors is only 35%–45% (Christie and Bowtell, 2017), with primary treatment resistance to standard-of-care platinum-taxane-based chemotherapy treatment occurring in 15%–25% of patients (Harries and Kaye, 2001). In addition, up to 75% of patients who initially respond to therapy, relapse and effective second line treatment therapies are lacking (Harries and Kaye, 2001; Cooke and Brenton, 2011; Brooks et al., 2019). Drug discovery approaches aiming to overcome this problem and improve treatment response of OC patients have relied heavily on *in vitro* techniques involving the growth of OC cell lines on tissue culture polystyrene, however, this leads to changes in cell morphology, phenotype, signaling and ultimately drug sensitivity (Baker and Chen, 2012; Brooks et al., 2019). In addition, it has been shown that established cell lines do not capture disease heterogeneity across patients and hence are lacking in clinical relevance (Gillet et al., 2011; Yee et al., 2022). The TME plays a crucial role in modulating tumor drug sensitivity in ovarian tumors (Yang et al., 2020). For example, the fallopian tube TME of primary HGSOC tumors is characterized by higher ECM deposition (especially laminin), dysplasia, loss of cellular polarity of fallopian tube epithelial cells, and release of several soluble factors that regulate ROS-induced stress, anoikis, invasion and cell growth (Cheng et al., 2012; Alsina-Sanchis et al., 2016; Huang et al., 2016; Micek et al., 2020). In addition, several studies linked CAFs to a poor prognosis in ovarian cancer patients, as they increase resistance to platinum-based drugs and accelerate tumor reoccurrence via upregulation of fibroblast activation protein alpha (FAP), which ultimately promotes tumor cell proliferation, invasion and migration via its gelatinase activity (Mhawech-Fauceglia et al., 2015). Further, a subpopulation of CAFs (CAF-S1) found in mesenchymal HGSOC tumors upregulates CXCL12, which plays a role in increasing the attraction, survival, and differentiation of immunosuppressive CD25+FOXP3+ T lymphocytes (Givel et al., 2018). Notably, infiltration of Tregs into advanced ovarian carcinomas is linked to poor clinical outcome and decreased survival. Besides Tregs, M2 macrophages are abundant in ovarian tumors and drive tumor progression, invasion, metastasis and angiogenesis, but also secrete immunosuppressive cytokines such as IL1R decoy, CCL17, CCL22 and IL10, which suppress the proliferation and function of cytotoxic T cells. While the ovarian TME is characterized with low infiltration of predominantly dysfunctional (exhausted) T cells, patients with high percentages of CD8 T cell infiltration showed prolonged survival compared to patients with classically ‘cold’ ovarian tumors (Yang et al., 2022). Collectively, these observations underscore the imperative for advancing drug screening approaches in ovarian cancer through the development of more physiologically relevant *in vivo* microenvironment models incorporating diverse components of the TME (Table 1).

**TABLE 1:** Microphysiological systems for tumor-stromal-immune study of ovarian cancer.

Cancer model	Reference	Key features	Cell types	Key findings
Ovarian	Dadgar et al. (2020)	• Single chamber with 19 microwells	PDX tumors of the HGSOC type	Superior spheroid formation and viability compared to large volume 3D cultures
		• Multi-chamber with 8 chambers		
Ovarian	Wan et al. (2023)	Microfluidic device with three parallel channels	ECs, fibroblasts, SKOV3, OV90 spheroids, CAR T cells	High level of tumor vascularization and recruitment of CAR T cells
Ovarian (metastasis)	Rizvi et al. (2013)	Microfluidic chip with three channels	OVCAR-5 cells	Increased EMT, motility, and tumor aggressiveness
Ovarian (metastasis)/Omentum	Ibrahim et al. (2022)	Microfluidic device with 4 channels	ECs, adipocytes, mesothelial cells, SKOV3/OVCAR3/OV90 cells	Enhanced tumor cell attachment and affects vessel formation
Ovarian (metastasis)	Saha et al. (2020)	Microfluidic device with 2 parallel chambers	HUVECs, platelets, A2780, HOMECs, OVCAR3	Influence on vascular luminal microenvironment and platelet extravasation
Ovarian (metastasis)	Saha et al. (2021)	Microfluidic device with three parallel microchannels	HOMECs, platelets, A2780/OVCAR3	Interaction through GPVI and galectin-3, impacting metastatic potential
Ovarian (metastasis)	Surendran et al. (2021)	Spheroids embedded in a microwell attached to channels	OVCAR-3, neutrophils	Induced NETs formation, tumor morphological changes, and invasion
Ovarian (metastasis)	Hachey et al. (2023a)	Microfluidic device with 1 cell chamber	ECs, lung fibroblasts, COV362	Perfusion through micro-vasculature

The increasing interest in utilizing physiologically relevant *in vitro* models to improve the drug development pipeline and explore more personalized treatment approaches in ovarian cancer has led researchers to combine cancer spheroids with 3D microfluidic approaches (Dadgar et al. (2020); Nath and Devi (2016); Cui et al. (2017); Gupta et al. (2022). Spheroids represent a useful tool for maintaining patient-specific tumors *in vitro* and functional and genomic similarities between primary serous OC tumor tissue or ascites and matched spheroids have been shown (Dadgar et al., 2020; Marrella, 2020; Velletri et al., 2022). For example, Dadgar et al. incorporated multiple patient-derived xenograft (PDX) tumors of the HGSOC subtype into microfluidic devices, where they observed enhanced spheroid formation, proliferation, and viability under microfluidic conditions compared to larger-scale 3D cultures or Matrigel (Dadgar et al., 2020). Importantly, phenotypic characteristics were maintained in the microfluidic cultures. Subsequently, the researchers used a multi-chamber microfluidic device, which allowed for serial seeding of chambers and independent, parallel perfusion, in order to test the response of each PDX spheroid to doxorubicin and gemcitabine. Thus, this microfluidic device offers a promising platform for drug testing and personalized therapy approaches if only limited tumor material is available and with the caveat that intercellular interactions and vasculature-mediated drug delivery are absent.

In an approach to improve tumor architecture, promote tumor-stromal interactions and promote tumor vascularization, Wan and colleagues developed a tumor spheroid model that allows for high levels of tumor vascularization via a living vasculature network and further allows elucidation of the role of fibroblasts within the TME (Wan et al., 2023). In fact, fibroblasts are described to play an important role in tumor vascularization *in vitro* by inducing angiogenic processes, however, it is unclear if the location of fibroblasts in the tumor spheroid is crucial for promoting vascularization. To answer this question, ovarian tumor spheroids formed by OV90 or SKOV3 cells, along with ECs and fibroblasts were co-seeded into a single-chamber microfluidic device. In total three types of spheroids were tested, with one type formed by tumor cells only, another type by the co-mixture of tumor cells and fibroblasts and the last group of spheroids by a sequential approach in which fibroblasts were added to the pre-formed tumor spheroid, leading to a higher concentration of fibroblasts on the spheroid outside. Interestingly, the tumor spheroids formed by the sequential approach led to the highest level of tumor vascularization, vessel perfusability and fibroblast area, which is indicative of complex microenvironmental remodeling processes and enhanced angiogenesis that typically occurs in in vivo vascularized tumors (Wan et al., 2023). In addition to replicating a vessel-rich TME by integrating fibroblasts as an essential stromal component, the researchers added a micropump to their system that allowed for the continuous re-circulation of mesothelin-directed CAR-T cells in order to evaluate their effectiveness against vascularized SKOV3 spheroids. Interestingly, CAR T cells were recruited to the tumor spheroids, extravasated from the tumor vessels and engaged in interactions with tumor cells; leading to a strong IFN-γ response and a higher density of dead cells in the tumor region (Wan et al., 2023). In summary, this pioneering approach represents one of the first endeavors to incorporate a living vasculature network, which not only influences but is also influenced by tumor cells, stromal cells, and immune cells, thereby showcasing its translational potential. Future studies are necessary to evaluate the precise intercellular interactions and how these signaling mechanisms influence responsiveness to therapeutic drugs or immunotherapy.

Metastasis of ovarian tumors is common. Current research suggests that HGSOC initiates in the fallopian tube, then metastasize to the ovaries and can then spread to secondary metastatic sites via lymphatic, hematogenous and transcoelomic routes, whereby the latter is the most frequent (Micek et al., 2020). Secondary metastatic sites can include the mesentery, the liver, the omentum or the peritoneum (Micek et al., 2020). Metastasis to the peritoneum is accompanied by an abnormal accumulation of ascitic fluid rich in cells and proteins, and this is associated with a poor median patient survival (Finkernagel et al., 2019; Huang and Tu, 2023). A critical step in the progression of ovarian cancer metastasis is the epithelial-to-mesenchymal transition (EMT), which is characterized by the loss of E-cadherin, cytoskeletal remodeling and changes in proliferation, motility and apoptosis (Ahmed et al., 2007; Moreno-Bueno et al., 2009; Datta et al., 2021). In order to better understand the physical and biological factors influencing ovarian cancer metastasis, Rizvi and colleagues developed a microfluidic platform that enables the investigation of fluidic forces as modulators for tumor metastasis (Rizvi et al., 2013). In particular, their microfluidic device enabled the formation of 3D micro-tumor nodules of OVCAR-5 cells on Matrigel under continuous flow. Interestingly, in comparison to static 3D cultures, the ovarian tumor nodules grown under flow exhibited morphological and phenotypical features indicative of increased EMT, such as an increase in epidermal growth factor receptor (EGFR) expression and activation, an increase in vimentin expression and the downregulation of E-cadherin expression. As the modulation of EMT biomarkers is a critical step in tumor metastasis, this model underlines the fact that hydrodynamic forces can drive disease aggressiveness. However, other physical, biochemical and cellular cues of the TME also significantly contribute to ovarian tumor metastasis.

On this note, Ibrahim and colleagues recognized the need to create a platform that allows for the elucidation of tumor-stromal interactions that are essential for the peritoneal dissemination of ovarian tumors (Ibrahim et al., 2022). Therefore, they developed an *in vitro* vascularized model of the human omentum and ovarian TME, whereby they used a microfluidic platform that included ECs, omental adipocytes and mesothelial cells (in various combinations) to study the effect of stromal cells on tumor cell attachment and growth, as well as the effect of cancer cells on vascular and mesothelial permeability. While the coculture of ECs and adipocytes resulted in micro-vessels closely resembling those present in the omentum, the addition of mesothelial cells significantly improved mimicking *in vivo* omental conditions, underlining the influence of the mesothelial-adipocyte signaling axis to alter vessel diameter, formation and permeability. Further, these interactions were shown to weaken mesothelial barrier function and permeability similar to *in vivo* conditions and also lead to modulations of collagen and fibronectin deposition in the microfluidic device. Importantly, these two ECM components are especially enriched in adipose tissue and the peritoneum (Shibata et al., 1997; Witz et al., 2001; Khan et al., 2009; Mori et al., 2014; Lin et al., 2016). Lastly, the invasion and growth into the peritoneum of three different ovarian cancer cell lines (SKOV3, OVCAR 3, OV90) was tested, where it was noted that the presence of stromal cells, such as sub-mesothelial ECs and adipocytes, could enhance the attachment of ovarian tumor cells to the mesothelium and also enhance tumor growth. While precise molecular signaling cascades need to be further evaluated, the value of this model is determined by its ability to assess ovarian tumor-stromal interactions in a physiologically relevant TME.

Platelets also interact with (circulating) tumor cells and promote tumor metastasis. Platelets can extravasate into the primary ovarian tumor and induce proliferation and resistance to chemotherapy (Cho et al., 2012; Stone et al., 2012; Hu et al., 2017). Since the mechanism of how platelets traffic and extravasate into the tumor is poorly understood, Saha and colleagues performed a proof-of-concept study showing that platelet extravasation can be modeled in the context of microfluidic chips (Saha et al., 2020). Their device consists of a tissue chamber that is lined with A2780 ovarian cancer cells and interfaced with a 3D endothelialized lumen, that can subsequently be perfused with human platelets (Figure 2, panel B). Upon perfusion, platelets adhered to cancer-interacting ECs, extravasated and colonized into tumors; mirroring *in vivo* observations. Further, the researchers were able to show that the increased expression of cytokines (IL-8, MCP-1, IL-6, and TNF-α) by the cancer cells led to endothelial adhesion junction degradation, increased gap formation and loss of endothelial barrier function that was accompanied by the activation of the Src/ERK/FAK signaling pathway and downregulation of the vascular barrier regulators VE-cadherin and VE-PTP (Saha et al., 2020). Interestingly, treatment of the ovarian tumor chips with atorvastatin reversed the overexpression of proinflammatory cytokines, normalized Src/ERK/FAK pathway signaling and led to enhanced barrier function in ECs. These findings were consistent across five patient samples of the HGSOC type, indicating the potential of atorvastatin as a vasculature-normalizing drug that limits platelet extravasation and indirectly influences tumor metastasis. In a subsequent study with an improved microfluidic chip design integrating a more physiological ECM, Saha et al. explored the effectiveness of a glycoprotein VI (GPVI) inhibitor to limit platelet extravasation (Saha et al., 2021). Their microfluidic chip revealed that the interaction of platelets and tumors occurs via glycoprotein VI and tumor galectin-3, a protein typically overexpressed in advanced stages of ovarian cancer. They also showed that the administration of GPVI led to reduced cancer proliferation, invasion and chemotherapy resistance. This highlights the therapeutic potential of platelet-targeting drugs in the context of limiting ovarian cancer metastasis and encourages further research to better understand the complex processes in the TME.

Less than a handful of ovarian cancer-based micro-fluidic chips incorporates immune cells, however, the TME plays an essential role in tumor initiation, development and therapy response (Surendran et al., 2021; Bouquerel et al., 2023). Neutrophils represent a significant portion of cells in the TME (Swierczak et al., 2015; Powell and Huttenlocher, 2016), and their abundance has been linked to adverse prognostic outcomes in cancer patients (Hiramatsu et al., 2018; Schiffmann et al., 2019; Yin et al., 2019; Kim et al., 2022). In their study, Surendran and colleagues aimed to investigate the effect of neutrophils on ovarian cancer cell invasion (Figure 2, panel C), whereby they used OVCAR-3 spheroids embedded in a collagen-covered microwell that is connected to a microfluidic channel designed to replicate an intact vascular structure (Surendran et al., 2021). Interestingly, upon the introduction of neutrophils into the microfluidic channel, the cells migrated through the collagen matrix towards the ovarian spheroids and were able to elicit in vivo-like neutrophil responses, such as tumor-mediated chemotaxis and NET formation. Subsequently, NET formation stimulated the elongation of OVCAR-3 cells, induced loss of circularity of tumor spheroids, enhanced tumor migratory behavior and induced tumor cell collective invasion into the collagen matrix in a highly directional manner. Notably, this process can be reversed via the drug-induced inhibition of NETs formation, potentially opening a new therapeutic avenue for limiting ovarian cancer metastasis in patients.

### 3.2 Pancreatic cancer

Pancreatic Ductal Adenocarcinoma (PDAC) is projected to become the second leading cause of cancer-related deaths by 2030, exhibiting a high mortality rate with a median survival time of less than 6 months and 5-year survival rates below 4% (Haque et al., 2021). Significantly, the aggressive progression of PDAC, characterized by immune evasion and therapeutic resistance, is heavily influenced by the TME (Balachandran et al., 2017). PDAC is distinguished by stromal desmoplasia and vascular dysfunction, which poses challenges to effective drug delivery. The high-density stroma of PDAC, comprised of cancer-associated fibroblasts (CAFs), adipocytes, immune cells, ECs, nerve cells, pancreatic stellate cells (PSCs), and extracellular matrix components (ECM) like collagen and hyaluronic acid, constitutes up to 90% of the tumor volume (Jacobetz et al., 2013; Poh and Ernst, 2021; Wu et al., 2021). Notably, an increased density of macrophages in the tumor serves as an independent prognostic factor in PDAC patients, correlating with an elevated risk of disease progression, recurrence, metastasis, and reduced overall survival (Poh and Ernst, 2021). Additionally, PDAC is marked by poor vascularization and abnormal leaky blood vessels, creating hypoxic conditions and elevated interstitial pressure (Jacobetz et al., 2013). These factors contribute to the formation of an immunosuppressive microenvironment, diminishing the efficacy of immunotherapies. While many existing *in vitro* PDAC models lack translational relevance due to their inability to capture essential aspects of PDAC complexity, there is a promising emergence of innovative platforms specifically designed to capture the unique tumor microenvironment of this disease (Table 2).

**TABLE 2:** Microphysiological systems for tumor-stromal-immune study of pancreatic ductal adenocarcinoma.

Cancer model	Reference	Key features	Cell types	Key findings
PDAC	Haque et al. (2022)	Device with 2 chambers separated by porous membrane, pump	Patient-derived PDAC organoids, PSCs and macrophages	Anti-stromal compounds enhance PDAC chemotherapy response in tumor chip
PDAC	Mollica et al. (2021)	Microfluidic device with 2 lateral channels and central chamber	ECs, PSCs, Panc-1 PDAC cells, and T cells	EC barrier decreases T cell infiltration into PSCs + Panc-1
PDAC	Lee et al. (2020)	Microfluidic chip with 4 connected channels	Panc-1 PDAC cells and macrophages	Interstitial flow and cancer cell signaling increases macrophage motility

Several research teams are currently combining two advanced *in vitro* technologies—organoid cultures and tumor-chip systems—to develop innovative organotypic models for improved patient stratification and therapeutic decision-making in PDAC (Narayanan et al., 2021; Osuna De La Peña et al., 2021; Geyer et al., 2023). Tumor-chip technology is pivotal in optimizing the use of sparse tissue obtained from clinically indicated endoscopic fine needle biopsies for PDAC tissue diagnosis, addressing challenges in precision medicine, such as limited access to surgical specimens due to a high percentage of unresectable cases (>80%) and the rapid progression of the disease (Narayanan et al., 2021). Despite the substantial resemblance of patient-derived PDAC organoids to the parent tumor, there is a growing awareness that current organoid culture techniques can be significantly enhanced. Organoids fail to replicate the intricate 3D PDAC architecture and interactions within the tumor stroma. Furthermore, static culture conditions result in organoids relying solely on passive diffusion for nutrient and oxygen intake, as well as waste product removal. As organoids grow larger, diffusive transport becomes inadequate to meet their increasing metabolic demands, ultimately hindering their growth. Moreover, the absence of dynamic flow conditions impedes the formation of morphogen gradients essential for replicating the mechanistic underpinnings of PDAC progression. Additionally, the accurate recapitulation of physiological barriers to the delivery of therapeutics and immune cells in PDAC is lacking.

To address these challenges, a chip-based biomimetic ductal TME was developed to replicate the microanatomy of PDAC (Bradney et al., 2020). The model consisted of a duct lined with pancreatic cancer cell epithelium embedded in a collagen matrix, forming a perfusable epithelial lumen that mimicked physiological biomechanical forces. Murine pancreatic cancer cells from two genetically engineered mouse models, representing common PDAC mutations, were utilized—one with KRAS and TP53 mutations and the other with KRAS and CDKN2A mutations, displaying distinct mesenchymal and epithelial phenotypes. This model facilitated the exploration of varied local invasion patterns in response to TGF-β1 based on genotype and phenotype, as well as the investigation of heterogeneous interactions among diverse cancer cell populations (Bradney et al., 2020). In a separate recent study, researchers pioneered the integration of microfluidics and dielectrophoresis to culture PDAC human cell lines in a cyclic olefin polymer chamber, creating a biomimetic platform named HepaChip (Beer et al., 2017). This platform enables the culture of PDAC spheroids under continuous flow conditions and accurately captures PDAC cell behavior, displaying growth characteristics closer to 3D spheroid cultures than traditional 2D cultures. Notably, preliminary experiments demonstrated the platform’s capability to predict PDAC cell responses to Cisplatin, showing an increased IC50 on-chip compared to 2D cultures and closer alignment to *in vivo* responses.

Recognition of the pivotal role of stromal cell populations on PDAC progression has led to the increasing integration of multiple cell types into microfluidic platforms. A humanized microfluidic model of the PDAC microenvironment, incorporating pancreatic stellate cells (PSCs) co-cultured with ductal adenocarcinoma cells embedded in collagen I and hyaluronic acid matrices, allowed for imaging of live cell-collagen interactions (Drifka et al., 2013). The study demonstrated the model’s potential to elucidate complex stroma-cancer interrelationships and evaluate therapeutic efficacy. Another study employed a multilayer PDAC-on-chip design to culture PDAC cells, PSCs, and fibroblasts in separate compartments. The findings revealed that fibroblasts altered their morphology to a myofibroblast-like shape and secreted increased amounts of IL-6 in the presence of cancer cells, potentially modeling the early steps of PDAC evolution (Sgarminato et al., 2023). The incorporation of vascularization into tumor-chips is a crucial strategy to mimic proper *in vivo* physiology, particularly for PDAC where high interstitial pressure leads areas of vascular collapse and poor perfusion. A recent study introduced a perfusable, 3D organoid-on-a-chip model to recapitulate the PDAC TME (Lai et al., 2020). Co-culturing patient-derived pancreatic cancer organoids and stromal fibroblasts within a perfusable vascular system successfully mimicked PDAC desmoplasia, demonstrating inhibitory effects on known chemotherapeutic treatments. This organoid-on-a-chip model may serve as a valuable tool for studying the interactions between cancer cells and the TME, providing a platform for personalized medicine in PDAC. In another study, a biomimetic 3D organotypic model unveiled a mechanism by which pancreatic cancer cells induce endothelial ablation, resulting in tumor-driven endothelial cell death and hypoxia (Nguyen et al., 2019). The study identified the activin-ALK7 signaling pathway as a key mediator of this endothelial ablation, suggesting a potential therapeutic strategy for pancreatic cancer by targeting this pathway.

Although these models show promise toward advancing our mechanistic understanding of tumor-stromal interactions in PDAC, there remains a shortage of models that effectively capture the intricate dynamics of the tumor-immune interface in PDAC. In recent advancements in PDAC immuno-oncology research, several innovative platforms have been developed to elucidate the intricate dynamics and biophysical properties of the tumor immune microenvironment (TME) to optimize drug responses. A PEG hydrogel-based platform that accurately mimics the pancreatic desmoplastic microenvironment was recently developed, offering an advanced model for studying PDAC and revealing the functional significance of laminin-integrin α3/α6 signaling in pancreatic organoid survival (Below et al., 2022). The inclusion of primary pancreatic fibroblasts and macrophages within the hydrogel created a more physiologically relevant microenvironment. Additionally, Haque and colleagues designed a two-chamber microfluidic device with continuous perfusion to replicate the PDAC TME (Haque et al., 2022). This involved utilizing patient-derived organoids and human stromal cells, specifically PSCs and macrophages. The study unveiled reciprocal interactions between neoplastic pancreatic organoids and stromal cells, which fostered synergistic growth. The platform proved valuable for drug testing, revealing the adjuvant effects of anti-stroma agents combined with chemotherapy in the intercellular PDAC organoid-based chip, in contrast to the monoculture response. This underscores the tumor-chip’s potential for identifying novel drugs targeting the stroma to enhance chemotherapy efficacy in PDAC. In a separate investigation, Lee and colleagues developed an integrated *in vitro* and *in silico* model to examine macrophage migration influenced by biochemical and mechanical factors in the tumor microenvironment (Figure 2D) (Lee et al. (2020). The model demonstrated that macrophages are attracted to regions of high interstitial fluid pressure, mediated by the chemokine CCL2, and can enhance tumor cell migration and invasion through matrix metalloproteinase secretion. This integrated approach provided valuable insights into the pivotal role of macrophages in tumor progression and metastasis.

To investigate T cell infiltration, an integral aspect of anti-tumor immune responses in PDAC, researchers developed a 3D *in vitro* PDAC-TME model (Mollica et al., 2021). This innovative model employs a three-channel microfluidic device wherein PDAC cells are cultured in a central collagen matrix, bordered by ECs mimicking blood vessels on one side and pancreatic stellate cells (PSCs) simulating the exocrine pancreas on the other. The model facilitated the observation and quantification of T cell infiltration across the vasculature, offering insights into how TME composition influences T cell migration. Notably, the presence of ECs significantly reduces T cell infiltration, underscoring the pivotal role of blood vessels in regulating T cell trafficking. In contrast, co-culturing PDAC cells with PSCs markedly increased T cell infiltration, emphasizing the influence of the TME on immune cell behavior. The model also allowed for the evaluation of T cell migration and cytokine production, providing valuable insights into the dynamics of T cell responses within the TME. Activated T cells exhibited a 50% increase in migration towards cancer cells compared to their non-activated counterparts. This 3D pancreatic tumor model may serve as a valuable platform for studying T cell infiltration and tumor-immune interactions, contributing to the development of effective immunotherapeutic strategies for PDAC. The combined results from these studies underscore the importance of advanced models in comprehending the intricate and immunosuppressive PDAC microenvironment, deciphering therapeutic responses, and advancing personalized medicine strategies.

### 3.3 Renal cell carcinoma

Kidney cancer ranks as the eighth most common cancer in the United States, with less than a 12% 5-year survival rate for cases diagnosed as metastatic disease (Heidegger et al., 2019). The majority (85%–90%) of kidney cancers originate from proximal tubular epithelial cells in the renal cortex and are termed renal cell carcinoma (RCC). Clear cell renal cell carcinoma (ccRCC), representing about 75% of RCC cases, exhibits nests of malignant epithelial cells with clear cytoplasm surrounded by an extensive, arborizing vasculature (Heidegger et al., 2019). Sporadic ccRCCs in humans often harbor inactivating mutations in the VHL tumor suppressor gene, leading to continuous stabilization of hypoxia-inducible transcription factors HIF-1α and HIF-2α (Shapiro et al., 2022). This results in metabolic reprogramming toward aerobic glycolysis and the secretion of factors, including VEGFA, that foster angiogenesis. While clear cell renal cell carcinoma (ccRCC) has traditionally been labeled as a ‘cold’ tumor, it does exhibit some degree of response to immune checkpoint inhibitors (ICIs), and current standard care involves the combination of ICIs and TKIs like pembrolizumab and axitinib (Pohl et al., 2022). However, a notable proportion of patients experience disease progression shortly after initiating systemic treatment. Additionally, outcomes from adjuvant ICI trials for localized ccRCC have not shown improvements in survival (Raghubar et al., 2023). These unsuccessful trials suggest that the types, states, and spatial distribution of tumor-infiltrating immune cells within the TME may not favor ICI treatment.

A contributing factor to therapeutic failure is the distinct immunological characteristics of ccRCC, including a high number of tumor-infiltrating T cells, which paradoxically correlates with a poor prognosis (Pohl et al., 2022). Interestingly, immune-excluded ccRCC tumors, unlike counterparts in other cancers, are linked to a more favorable prognosis. Tumor-infiltrating T cells often express inhibitory checkpoint proteins and undergo exhaustion, particularly in advanced ccRCCs (Heidegger et al., 2019). T cell exhaustion in advanced ccRCC involves interactions with immunosuppressive M2 macrophages, creating a positive feedback that promotes tumor progression (Heidegger et al. (2019). Non-immune cells, by secreting factors like FGF-2, further support tumor growth. Yet recapitulating the dynamic and complex nature of microenvironmental cell-cell interactions in RCC *in vitro* remains a challenge in model development. To address this objective, multiple research groups are developing tumor chips designed to enable the co-culture of RCC cells within a TME that is both structurally and physiologically relevant (Table 3). In a recent investigation, a metastasis-on-a-chip model was crafted to replicate the progression of kidney cancer cells in the liver (Wang et al., 2020). Recognizing the vital role of organ-specific extracellular matrix (ECM) in metastatic cancer, the study employed a decellularized liver scaffold to create a biomimetic liver-specific ECM. This ECM was then used to seed HepLL liver cells and Caki-1 RCC cells. The model facilitated the evaluation of the anti-cancer drug 5-FU against metastatic kidney cancer cells, revealing that a 5-FU-loaded poly (lactide-co-glycolide) (PLGA)-poly (ethylene glycol) (PEG)-based nanoparticle delivery system exhibited greater efficacy than free 5-FU in eliminating Caki-1 cells.

**TABLE 3:** Microphysiological systems for tumor-stromal-immune study of renal cell carcinoma.

Cancer model	Reference	Key features	Cell types	Key findings
RCC	Miller et al. (2023)	Chip with 2 media channels flanking a central matrix region	RCC cell lines A498 and RCC4, CAR-T cells directed to ROR1	Increased stiffness of collagen matrix limits CAR-T cell infiltration

Crucially, microfluidic systems have shown promise in replicating the dense tumor neovascularity observed in RCC, mirroring key features of tumor-associated vessels, including aberrant organization, excessive angiogenic sprouting, and increased vessel permeability. In a recent study by Virumbrales-Munoz et al., a 3D *in vitro* co-culture model of clear cell renal cell carcinoma (ccRCC) was developed, comprising induced pluripotent stem cell-derived vascular ECs (iECs) and ccRCC epithelial cancer cells (A498 or Caki-2) (Virumbrales-Muñoz et al., 2022). The model faithfully recreated essential aspects of the ccRCC microenvironment, including angiogenesis, hypoxia, and metabolic reprogramming, allowing the study of dynamic interactions between tumor and ECs. In a proof-of-concept study, the model identified cabozantinib as a potential therapeutic agent for ccRCC, inhibiting tumor growth and metastasis. This model holds promise for personalized medicine applications, including testing the effects of anti-angiogenic drugs and immunotherapies, as it can be generated using patient-derived induced pluripotent stem cells (iPSCs).

Virumbrales-Munoz and colleagues further employed a microfluidic device to generate patient-specific blood vessel models, effectively replicating phenotypic differences between ccRCC tumor-associated and normal vessels (Virumbrales-Muñoz et al., 2020). Transcriptomics revealed a pro-angiogenic phenotype in tumor-associated vessels, showing patient-specific responses. Online databases identified alternative drugs targeting these vessels, and two drugs (nintedanib and sirolimus) were assessed. Nintedanib, a multi-tyrosine kinase inhibitor, decreased sprout number and length, enhancing vessel permeability. Sirolimus, a specific mTOR inhibitor, exhibited lower effectiveness and only restored permeability at high doses, despite increased cell toxicity. The results underscore the capability of microfluidic vessel models for functional drug testing, enabling personalized screening and patient stratification in ccRCC. In a 2018 study, primary human ccRCC cells from six donors were combined with normal-adjacent renal cortex cells flanking a human EC-lined vessel in a vascularized, flow-directed, 3D culture system known as ‘ccRCC-on-a-chip’ (Miller et al., 2018). This innovative co-culture model, incorporating ccRCC cells and HUVECs, faithfully replicated angiogenic features observed in ccRCC tumors, such as the formation of vascular networks and sprouting angiogenesis. Primary cultures from ccRCC tumors exhibited an angiogenic phenotype, marked by increased expression of angiogenic factors like ANGPTL4, PGF, and VEGFA. The application of a neutralizing antibody against ANGPTL4 signaling reduced sprouting angiogenesis in the ‘ccRCC-on-a-chip’, suggesting the involvement of ANGPTL4 in ccRCC angiogenesis. This system holds promise for assessing cancer-vascular interactions, evaluating antitumor treatments, and, in the future, investigating immune cell interactions within the ccRCC TME that impact responses to immunotherapy.

In a subsequent investigation, Miller et al. employed a simplified version of the ‘ccRCC-on-a-chip’ platform lacking vasculature to assess RCC tumor migration, invasion, and responses to therapies, including cell-based immunotherapies (Miller et al., 2023). The chip’s design comprises three sites, each with a central matrix region for collagen injection, flanked by two parallel media channels for gravity-based flow and administration of therapeutic compounds or cells, such as T cells. This 3D model facilitated the simultaneous observation and quantification of spheroid cell death and collective migration in response to a biomimetic collagen ECM. Gene expression profiling highlighted significant differences between cells cultured in 3D and 2D, with 3D cultures exhibiting an expression profile closer to primary tumors. GSEA revealed multiple enriched pathways associated with these differentially expressed genes, including hypoxia-inducible genes. Twelve genes, significantly upregulated in both RCC cell lines (A498 and RCC4) and primary RCC vs normal tissues in TCGA, included the same three proangiogenic genes previously identified (VEGFA, ANGPTL4, and PGF). Interestingly, cytotoxicity induced by the chemotherapeutic drug bortezomib was observed at lower concentrations in 3D compared to 2D cultures. The assay demonstrated feasibility in testing cellular-based therapeutics, exposing challenges in T-cell penetration of the collagen ECM, a phenomenon not measurable in traditional 2D cytotoxicity assays. Specifically, the assessment of antigen-receptor dependent killing of RCC spheroids expressing ROR1, a tumor antigen under clinical trial investigation, unveiled limited T-cell infiltration into the high-density collagen matrix compared to low-density collagen. Collectively, these studies underscore the relevance of tumor chips for advancing personalized immuno-oncology efforts in immunologically ‘cold’ RCC tumors.

### 3.4 Prostate cancer

Prostate cancer (PCa) is the second leading cause of cancer-related death in men in the United States and is projected to claim the life of more than 35,000 patients this year. The incidence and mortality rate correlates with advancing age and individuals over 65 years old account for approximately 60% of cases (Howlader et al., 2016; Siegel et al., 2024). Initially, the growth and survival of PCa cells rely on androgens, particularly testosterone, which binds to the androgen receptor (AR) and orchestrates the expression of numerous proteins involved in cell proliferation and/or mechanisms to evade apoptosis. The standard therapy approach for PCa is androgen deprivation therapy (ADT), however, approximately 10%–20% of PCa patients develop castration resistant prostate cancer (CRPC); rendering ADT ineffective and resulting in a dismal clinical prognosis with a median survival time of only 9–13 months for metastatic CRPC (Kirby et al., 2011). Tumorigenesis and therapy resistance development of prostate cancer is driven by changes in the TME (Ge et al., 2022). For example, MDSCs present in the TME have been shown to release IL23, which activates STAT3-RORγ signaling to drive AR transcription and castration resistance (Calcinotto et al., 2018). In addition, the immunosuppressive prostate TME is characterized by increased TGF-β expression by prostate tissue, poor cytolytic activity of NK cells and the presence of regulatory T cells (Sfanos et al., 2008; Pasero et al., 2016; Ahel et al., 2019; Ge et al., 2022; Ju et al., 2022). Paradoxically, the presence of neutrophils is associated with poor prognosis and tumor infiltration with CD4^+^ T cells has been linked to tumor progression and metastasis (Hu et al., 2015; Wu et al., 2020). In addition, mast cells contribute to increased chemotherapy and radiotherapy resistance (Xie et al., 2016), while tumor infiltrating CD8^+^ T cells are–similar to ovarian cancers or other ‘hot’ tumors–associated with improved clinical outcome (Yanai et al., 2021; Yang et al., 2021). Further, heterogeneous macrophage populations, consisting of both protumorigenic M2 macrophages producing high amounts of reactive oxygen species, IL12 and IL10 and antitumorigenic M1 macrophages, can be found in the prostate TME (Cortesi et al., 2018; Ge et al., 2022). Cellular interactions between these tumor-associated macrophages and invariant NK (iNK) cells can delay prostate cancer progression (Cortesi et al., 2018). Furthermore, hypoxia-dependent signaling can lead to mesenchymal-epithelial transition, promoting mesenchymal reprogramming and neuroendocrine transdifferentiation processes in prostate tumor cells (Lu and Kang, 2010; Davies et al., 2018). Lastly, CAFs present in the TME can orchestrate several pathways leading to increased tumorigenesis of prostate tumors, such as the recruitment of inflammatory immune cells (i.e., mast cells, eosinophils, Th2 cells), indirect recruitment of tumor-promoting macrophages, promotion of mesenchymal-epithelial transition processes and the remodeling of the extracellular matrix (Giannoni et al., 2010; Vickman et al., 2020; Ge et al., 2022; Bedeschi et al., 2023). Collectively, these observations underscore the imperative for advancing our understanding of prostate cancer through the development of physiologically relevant *in vitro* systems, that allow for the integration of multiple TME components to eventually offer better treatment approaches to tackle the ‘cold’ tumor microenvironment of prostate cancer patients (Table 4).

**TABLE 4:** Microphysiological systems for tumor-stromal-immune study of prostate cancer.

Cancer model	Reference	Key features	Cell types	Key findings
Prostate	Padmyastuti et al. (2023)	Chip with 2 independent circulations feeding two culture chambers	LNCaP or PC3 spheroids	MPS promote important physiological and morphological changes of prostate cancer cells and maintain PSA expression
Prostate	Jiang et al. (2024)	Device consisting of two stacked microchannels that are separated by a porous membrane	benign human prostate stromal cells, EMP or C4-2 or 22Rv1 tumor cells, primary human prostate basal epithelial cells	Prostate tumor cells drive the conversion of normal fibroblasts into CAFs, which promote tumor invasion via the secretion of TGF-β
Prostate	Chakrabarty et al. (2022)	Device with a tissue chamber that is separated from a microchannel by a parous membrane	Prostate tumor slices from PC82 PDX model	For assessing patient treatment responses using prostate tumor tissue slices
Prostate	Pandya et al. (2017)	Platform with integrated electrical microsensors	DU145 cancer cells	Real-time analysis tool for chemotherapeutic responses
Prostate	An et al. (2014)	Automated microfluidic system with 64 individually targetable cell culture chambers	PC3 cancer cells	Establishment of a high throughput drug screening system allowing for rapid and resource-saving drug testing
Prostate	Yu et al. (2019)	Reconfigurable microfluidic system consisting of individual, stackable layers	HUVECs, THP-1 (monocytes), nHDFs, LNCap or C4-2 prostate cancer cells	Prostate cancer cells polarize macrophages into pro- and anti-inflammatory phenotypes that signal distinctly to ECs
Prostate (metastasis)	Verbruggen et al. (2021)	Device with two parallel cell chambers separated by a porous membrane	MLO-Y4 cells osteocyte-like cells, PC3 or LNCaP cancer cells	Exerting mechanical fluid shear on osteocyte cells has no effect on PC3 proliferation, but stimulates their invasion and migration
Prostate (metastasis)	Hsiao et al. (2009)	Device with two microchannels separated by a semi-permeable membrane	HUVECs, osteoblasts, PC3 spheroids	Coculture of PC3, osteoblasts and endothelial cells turned PC3-based spheroids towards a more quiescent state

Padmyastuti and colleagues aimed to develop a basic microfluidic prostate cancer model capable of recreating the epithelial nature and PSA and miRNA secretion profiles of prostate cancer cells (Padmyastuti et al., 2023). They manufactured a chip containing two culture chambers that experienced pulsatile flow, leading to the contraction of the ECM hydrogel in each perfusion cycle and enabling the formation of LNCAP spheroids (androgen-sensitive) and supporting the growth of PC3 cells (androgen-insensitive). Interestingly, both cell lines cultured under dynamic flow conditions exhibited increased polarity and enhanced epidermal growth factor receptor (EGFR) surface expression compared to 2D cultures. Further, altered gene expression profiles, including the upregulation of adhesion molecules (i.e., EPCAM) and cytokeratins (i.e., CK5 and CK19) allowing resistance to mechanical stress, could be observed for LNCaPs in the MPS platform. In addition, the expression of several microRNAs (miRs) identified as potential biomarkers for the early detection of CRPC (Arrighetti and Beretta, 2021; Rönnau et al., 2021), including miR-3687 and miR-205 were altered in the microfluidic devices containing LNCaPs. In sum, this approach demonstrated that external stimuli, like flow, can promote essential physiological changes in prostate cancer cells, as well as changes in microRNA expression profiles. However, future research incorporating a more complex TME is necessary to improve physiological relevance and allow for a better understanding of the role of microRNAs as biomarkers for early prostate cancer diagnosis.

In an attempt to create a 3D *in vitro* model that can recapitulate physiologically relevant stromal-epithelial crosstalk of the prostate, Jiang and colleagues embedded benign human prostate epithelial (BHPrE) cells and benign human prostate stromal (BHPrS) cells into a microfluidic device, featuring two vertically arranged microchannels divided by a porous polyester membrane (Jiang et al., 2019). This device enabled the long-term *in vitro* coculture of epithelial and stromal cells, enabled paracrine and endocrine crosstalk and ultimately stimulated the differentiation of basal epithelial cells into luminal secretory cells. Based on this model, Jiang et al. developed a prostate-cancer-on-a-chip model, whereby human prostate cancer cells from 3 cell lines (EMP, C4-2 or 22Rv1) and fibroblasts were cocultured on opposite surfaces and subjected to flow (Jiang et al., 2024). Interestingly, the prostate tumor cells were able to drive the conversion of normal fibroblasts into CAFs, characterized by strong expression of aSMA and COL1A1, downregulation of AR and elongated morphology (Jiang et al., 2024). In addition, the tumor invasion into the stroma was evaluated, using a slightly modified device design that enabled the cells to cross the porous membrane. In fact, both stromal CAFs and tumor cells migrated into the neighboring channel, suggesting that the stromal compartment plays a tumor invasion-promoting role via the secretion of TGF-β. In sum, this prostate-cancer-on-a-chip model serves as a useful tool to dissect tumor-stromal mediated mechanisms of tumor development and progression. However, it fails to incorporate other essential cellular cues of the TME, such as immune cells, and does not evaluate the effect of tumor-stromal interactions on chemotherapy outcome or resistance development.

One study that includes the presence of immune cells as an essential TME component, has been conducted by Yu and colleagues (Yu et al., 2019). The researchers developed a versatile microfluidic system that enables the assembly of 3D tissue models by stacking individual layers of pre-conditioned microenvironments to allow for multicellular interactions. Specifically, this model was used to examine the prostate cell-mediated differentiation of macrophages, as well as macrophage-facilitated angiogenic processes. Accordingly, TH1 monocytes, epithelial cells and C4-2 (androgen-independent) or LNCaP tumor cells were prepared in separate layers. Subsequently, TH1 monocytes were stacked onto the prostate cancer cell layer, leading to the organotypical differentiation of TAMs (od-TAMs). Interestingly, both prostate cancer cell lines were able to polarize macrophages, however, LNCaPs expressed higher levels of CXCL10 leading to macrophages of the M1 phenotype and C4-2 cells expressed higher levels of IL10; skewing the macrophage population towards an M2 phenotype. Besides confirming gene expression differences between both tumor lines, functional differences were evaluated by stacking the prostate tumor-induced od-TAMs onto a layer containing cocultured fibroblasts and ECs to assess angiogenesis. Notably, endothelial networks showed significant differences when od-TAMs were present, underlining the fact that sequential signaling events in the TME orchestrate distinct cellular responses and functional outcomes. Further, Yu and colleagues were able to demonstrate that the *in vivo* recruitment of monocytes from the blood into the tumor by the prostate tumor cells can be replicated in their device. Lastly, this model was used to evaluate tumor heterogeneity of prostate cancer tissue samples from patients, whereby patient-specific od-TAMs were generated and transcriptional changes were assessed. In sum, this model provided a glimpse into the multicellular interactions occurring in the TME and highlighted the significance of spatiotemporal specificity in intercellular signaling for effective, patient-specific therapeutic intervention.

Thinking about personalizing the treatment strategy for prostate cancer patients and circumventing potential chemotherapy resistance development, Chakrabarty and colleagues developed a microfluidic platform in which they can incorporate (hydrogel-immobilized) prostate cancer tumor slices derived from PC82 PDX models into a tissue chamber that is separated from an underlying microfluidic channel by a porous membrane (Chakrabarty et al., 2022). Interestingly, the prostate cancer tumor slices maintained their viability, morphology, proliferation potential and gene expression profiles in the microfluidic device for 7 days, allowing for the assessment of the chemotherapeutic sensitivity of the tumors. The administration of apalutamide resulted in decreased AR expression, an increase in apoptotic cells and a significant decrease in proliferation, which replicates the effect of antiandrogen treatment in *ex vivo* cultures and *in vivo*. Hence, this model recapitulates *in vivo* prostate-cancer specific drug responses and paves the way for more patient-specific treatment strategies by incorporating primary tumor material. Following a similar approach, Pandya and colleagues aimed to improve the speed by which the chemotherapy response of prostate cancer cells can be determined in order to give patients the opportunity of faster therapeutic interventions (Pandya et al., 2017). They designed a microfluidic platform that contains microsensors that are able to measure the electrical response of DU145 prostate cancer cells, embedded in a 3D ECM, to chemotherapeutic drugs. While low concentrations of carboplatin had no effect on DU145 cells, higher concentrations led to a decrease in viability, confirming that the platform can distinguish between drug-susceptible and drug-resistant prostate cancer cells. Remarkably, the effect of chemotherapeutic treatment could be measured in less than 12 h. Besides this approach to optimize the speed and capacity for more individualized therapy approaches, An and colleagues have reported on the development of a fully automated high throughput drug screening system that incorporates 64 individually addressable cell culture chambers, where sequential and combinatorial concentrations of two drugs can be mixed and tested simultaneously (An et al., 2014). They demonstrate the utility of this system via investigating the efficacy of combinatorial treatment with tumor necrosis factor-alpha related apoptosis inducing ligand (TRAIL) and curcumin on PC3 prostate cancer cells. While this highlights the potential to screen multiple drugs and concentrations with limited (patient) cell and drug material, it clearly highlights a trend towards more simplistic modalities aiming to predict therapeutic responses that, *in vivo*, are dependent on a variety of factors.

A common complication of prostate cancer is metastasis to the bone tissue, which occurs in about 10% of patients with primary disease and 80% of patients with advanced, late-stage disease (Berruti et al., 2000; Bubendorf et al., 2000). To establish metastatic lesions in the bone marrow prostate cancer must adapt to a microenvironment that is primarily dominated by bone cells, such as osteocytes (Verbruggen et al., 2021). While osteocytes are key players in healthy bone remodeling and regulate effector cells such as osteoblasts and osteoclasts (Schaffler et al., 2014; Van Der Woude et al., 2017), their role in cancer progression is poorly understood. To address this knowledge gap, Verbruggen and colleagues used a microfluidic chip, enabling the coculture of osteocyte cells and prostate cancer cells in 2 cell chambers separated by a porous membrane. In this they aimed to elucidate the effects of osteocyte mechanical stimulation on the behavior of PC3 prostate cancer cells (Verbruggen et al., 2021). Interestingly, exerting mechanical fluid shear on osteocyte cells had no effect on PC3 proliferation, however, it did stimulate their invasion and migration. This demonstrates the important role of osteocytes in regulating prostate cancer cell behavior, underlining the need to incorporate this cellular cue in future *in vitro* models for physiological disease modeling. Besides osteocytes, osteoblasts present on the surface of bones play an important role in prostate cancer invasiveness (Zhang et al., 2009). To better understand the cellular interactions of prostate cancer and osteoblasts, Hsiao and colleagues designed a microfluidic chip that allowed the coculture of PC3, osteoblasts and ECs. (Hsiao et al., 2009). While their tri-cellular coculture system decreased the proliferation rate of PC3 cells in spheroids in comparison to 2D triculture and turned them towards a more quiescent state, viability was maintained; indicating that their microfluidic system was recapitulating the *in vivo* growth behavior of metastatic prostate cancer cells. Nevertheless, future research will need to evaluate the individual role of endothelial cell and osteoblast signaling on prostate cancer cell behavior in order to identify targetable pathways that might limit prostate cancer metastasis to the bone or other secondary organs. In addition, considering the fact that the TME of prostate cancer is enriched in a variety of immune cells including mast cells or macrophages, the development of a model that incorporates these cells is greatly needed.

## 4 Discussion

Integral to cancer development and therapeutic responses is the immune contexture, defining the spatial distribution and function of diverse immune components within the tumor (Fridman et al., 2017). Hence, there is an increasing demand for human model systems with high clinical predictability in immuno-oncology, ones that can replicate the tumor-immune diversity of each individual’s cancer in a context-specific manner. Simultaneously, developing human model systems for cancer immunotherapy presents a challenge, requiring the recapitulation of necessary tissue cell diversity, three-dimensional architecture, and physiological function to assess the pharmacological and toxicological effects of complex immunotherapeutic regimens. In this review, we outlined the state-of-the-art in the utilization of advanced 3D *in vitro* models and engineering techniques to enhance our understanding of the roles played by immune cells in the TME. These models have revealed mechanisms through which cancerous and stromal cells in the immunologically ‘cold’ TME can hinder the effective elimination of tumors by the immune system. This insight contributes to the identification of novel biological targets for drug development and personalized medicine. Furthermore, 3D microfluidic models serve as a platform for investigating the mechanistic interactions between tumor cells and immune cells, providing capabilities for high-resolution imaging and quantification of cell interactions (Park et al., 2019a; Chernyavska et al., 2023). These models offer the unique ability to decouple, and precisely manipulate, variables such as hypoxia, matrix stiffness, and flow.

While a minority of cancer patients exhibit remarkable responses, the majority do not respond to immunotherapeutic treatments, including immune checkpoint inhibitors (Ren et al., 2022). This emphasizes the necessity to develop strategies for understanding the contextual factors contributing to interpatient heterogeneity and addressing the unpredictable response rates. This challenge is particularly pronounced in immunologically ‘cold’ tumors, which pose difficulties in treatment and are associated with a poor prognosis (Bonaventura et al., 2019b; Wang et al., 2023). Hence, it is imperative to establish patient-specific models integrating tumor, stromal, and immune cells obtained from individual patients to encompass the substantial variability in disease states leading to therapeutic challenges. Achieving this goal necessitates the establishment or availability of annotated biobanks containing viable cancer cells and corresponding immune cells from cancer patients. Additionally, researchers must integrate various state-of-the-art technologies in cell and organoid culture, MPS, biofabrication, bioengineering, and ‘omics’ approaches (e.g., next-generation sequencing and mass spectrometry) into their platforms. The incorporation of single-cell RNA sequencing and spatial transcriptomics technologies will further contribute to a more precise understanding of the immune contexture within each patient’s tumor. By revealing the connections between tumor molecular profiles and the immune cell landscapes, tumor chips can play a crucial role in identifying predictive markers, thereby guiding personalized immunotherapy and enhancing treatment responses for patients.

As combination strategies for treating immunologically ‘cold’ tumors become more prevalent, there will be an equally important need for concurrent safety evaluations alongside efficacy testing, addressing ‘on-target, off-tumor’ and other toxicological effects. Despite their general tolerability, immune checkpoint inhibitors (ICIs) may induce cytokine storms in patients, and T cell-engaging bispecific antibodies and TCR/CAR-T engineered T cells, contingent on the target, could result in significant off-target tissue destruction, leading to associated morbidity and mortality (Sterner and Sterner, 2021; Johnson et al., 2022). Several groups are now working toward assessing these safety risks using microphysiological systems. In a recent investigation, researchers verified the efficacy of two human immunocompetent Organs-on-Chips—specifically, an Alveolar-Lung Chip and a Colon and Duodenum Intestine-Chip (Kerns et al., 2021). These chips successfully simulated the off-tumor toxicity associated with T cell-engaging bispecific (TCB) antibodies targeting FOLR1 and CEA. Importantly, they provided valuable insights into determining doses that effectively target tumor cells while sparing healthy tissues. Similarly, another study utilized a kidney organoid-on-chip model to test the off-target effects of a bispecific antibody targeting the RMF peptide, derived from the intracellular tumor antigen Wilms’ tumor 1 (WT1) (Kroll et al., 2023). The findings demonstrated that the TCB could induce cell death in kidney cells expressing the target antigen, with the extent of kidney cell killing dependent on the concentration and duration of TCB exposure. These studies underscore the capability of MPS models to evaluate safety in tissues prone to adverse effects from immunotherapeutic interventions. In the future, these organotypic platforms might serve as diverse metastatic organs for examining the immune system’s role in the premetastatic niche and its contribution to facilitating metastasis (Jouybar et al., 2023).

Despite these advances, there remains a scarcity of microphysiological systems designed to advance our understanding of the tumor-immune microenvironment in cancer progression. While some approaches focus on deciphering the interplay between physical cues of the microenvironment and tumor progression, others aim to integrate living tumor vasculature into their models or explore intercellular interactions between tumor-endothelial, tumor-stromal, and tumor-immune cells (Liu et al., 2021; Shelton et al., 2021). However, many of these approaches lack the complexity needed for vasculature-mediated delivery of drugs, platelets, and/or immune cells, along with assessing comprehensive tumor-stromal, tumor-immune, and immune-stromal interactions. Surprisingly, few microfluidic chip studies exist for immunologically ‘cold’ tumors, especially those focusing on T cell modulation. Fibrotic and desmoplastic stroma in the ‘cold’ tumor TME presents a challenge for T-cell penetration into the dense ECM, a phenomenon not captured by traditional 2D cytotoxicity assays. Improving T-cell infiltration to enhance interaction with tumor cells is crucial for boosting the efficacy of immunotherapies against solid tumors. Additionally, there is a need for further investigation into the mechanisms of T-cell activation, as existing studies on antigen presentation in cancer or models of the lymphatic system and lymph nodes are limited, primarily focusing on immunologically ‘hot’ tumors (Moura Rosa et al., 2016; Shim et al., 2019; Sun et al., 2019; Shanti et al., 2021). Therefore, future research should address this gap and investigate the role of T cell modulating immunotherapies, in combination with TME normalizing strategies, to overcome the immunologically ‘cold’ tumor phenotype.

In summary, the development of microfluidic devices aiming to model immunologically ‘cold’ cancers is still in its early stages, as the majority of models lack essential cellular and acellular components of the TME, lack physiologically relevant vascularization, and fail to enable complex cellular crosstalk. Only a limited number of models incorporate relevant stromal cells to facilitate the dissection of tumor-stromal interactions. Understanding the cellular cross-talk among tumor, immune, and stromal cells is crucial for exploring the value of immunotherapies in patients with immunologically ‘cold’ tumors. To achieve this objective, collaborative efforts involving basic and translational scientists, engineers, clinicians, oncologists, and surgeons are essential for advancing the field. However, the adoption of this technology faces challenges, including addressing concerns about limited cell sourcing, expanding accessibility to non-expert users, and obtaining regulatory approvals to fully leverage tissue chip data for precision medicine initiatives (Low and Tagle, 2018). Despite ongoing challenges in the widespread adoption of intricate biofabrication protocols and increasing throughput for tumor chip applications, commercialization efforts within the field may help alleviate these obstacles. While simplified drug testing platforms focusing solely on cancer cells exist, overlooking other elements in the TME, more complex model systems with an integrative approach are imperative. Developing such systems is crucial for exploring new therapeutic possibilities, particularly for cancer patients with tumors exhibiting an immunologically ‘cold’ phenotype.
